# Microgeographic differentiation in thermal performance curves between rural and urban populations of an aquatic insect

**DOI:** 10.1111/eva.12512

**Published:** 2017-08-02

**Authors:** Nedim Tüzün, Lin Op de Beeck, Kristien I. Brans, Lizanne Janssens, Robby Stoks

**Affiliations:** ^1^ Evolutionary Stress Ecology and Ecotoxicology University of Leuven Leuven Belgium

**Keywords:** countergradient variation, faster–slower model, growth‐survival trade‐off, thermal evolution, thermal reaction norms, time stress, urban ecology, urban heat islands

## Abstract

The rapidly increasing rate of urbanization has a major impact on the ecology and evolution of species. While increased temperatures are a key aspect of urbanization (“urban heat islands”), we have very limited knowledge whether this generates differentiation in thermal responses between rural and urban populations. In a common garden experiment, we compared the thermal performance curves (TPCs) for growth rate and mortality in larvae of the damselfly *Coenagrion puella* from three urban and three rural populations. TPCs for growth rate shifted vertically, consistent with the faster–slower theoretical model whereby the cold‐adapted rural larvae grew faster than the warm‐adapted urban larvae across temperatures. In line with costs of rapid growth, rural larvae showed lower survival than urban larvae across temperatures. The relatively lower temperatures hence expected shorter growing seasons in rural populations compared to the populations in the urban heat islands likely impose stronger time constraints to reach a certain developmental stage before winter, thereby selecting for faster growth rates. In addition, higher predation rates at higher temperature may have contributed to the growth rate differences between urban and rural ponds. A faster–slower differentiation in TPCs may be a widespread pattern along the urbanization gradient. The observed microgeographic differentiation in TPCs supports the view that urbanization may drive life‐history evolution. Moreover, because of the urban heat island effect, urban environments have the potential to aid in developing predictions on the impact of climate change on rural populations.

## 1 INTRODUCTION

Urbanization is rapidly increasing worldwide (Seto, Güneralp, & Hutyra, 2012), and the differences in environmental conditions between urban and rural areas have a major impact on the ecology and evolution of species (Alberti, 2015; Alberti et al., 2017; Parris, 2016). A key environmental difference is the considerably higher mean temperature in urban areas (“urban heat islands”; Gaston, Davies, & Edmondson, 2010), leading to extended growing seasons (Somers et al., 2013; Yang, Tian, & Chen, 2013; Zipper et al., 2016) and causing shifts in phenology (Neil & Wu, 2006; Townroe & Callaghan, 2014). Yet, surprisingly few studies looked at trait differentiation in response to urban heat islands, and we lack evidence whether these differences are genetic (reviewed in Chown & Duffy, 2015; Diamond, Dunn, Frank, Haddad, & Martin, 2015; but see Brans et al., 2017; Brans et al., 2017). Considering that the temperature difference between urban and rural areas fall frequently within the range of the expected temperature increases by 2,100 under IPCC (2013) scenarios, adaptation to urban environments can inform on the impact of climate change on organisms (Chown & Duffy, 2015; Youngsteadt, Dale, Terando, Dunn, & Frank, 2015; Youngsteadt, Ernst, Dunn, & Frank, 2017).

Thermal performance curves (TPCs), the continuous reaction norms of organismal performance traits (e.g., growth rate) in response to temperature, are useful tools for the study of differentiation in thermal responses (Sinclair et al., 2016; Stinchcombe & Kirkpatrick, 2012). The spatial differentiation of TPCs to compensate differences in local temperatures may take three not mutually exclusive adaptive patterns (Angilletta, 2009; Yamahira & Conover, 2002): (i) The TPCs may be shifted horizontally (“hotter–colder” model), with the thermal optimum being higher in warm‐adapted genotypes than in cold‐adapted genotypes as predicted by theory (Gilchrist, 1995; Lynch & Gabriel, 1987). (ii) The TPCs may be shifted vertically (“faster–slower” model), with cold‐adapted genotypes having a higher performance than warm‐adapted genotypes at all temperatures (for continuous thermal spatial gradients referred to as countergradient variation) (Conover, Duffy, & Hice, 2009; Conover & Schultz, 1995). Under countergradient variation, genetic and environmental effects oppose each other to produce similar performance in the natural populations along a thermal gradient. (iii) Based on the suggested trade‐off between maximal performance and thermal breadth, the TPCs may differ in their width (“generalist–specialist” model), with the performance breadth being narrower in genotypes with higher maximum performance than in genotypes with lower maximum performance. It is theoretically predicted that genotypes adapted to high compared to low temperature variations would have broader TPCs (Gilchrist, 1995; Lynch & Gabriel, 1987), yet studies have provided mixed empirical support (Angilletta, 2009). While “faster–slower” differentiation is not predicted by optimality models of thermal evolution (Gilchrist, 1995; Lynch & Gabriel, 1987), it is often documented for growth rate where it is believed to be driven by time constraints as the increased growth rates in colder environments compensate for the shorter growing season (Conover et al., 2009).

We here focused on geographic differentiation in TPCs for growth rate, a commonly used performance trait (Angilletta, 2009), between replicated urban and rural populations of an aquatic insect using a common garden rearing experiment with a range of temperatures. While urbanization gradients correspond to temperature gradients (Parris, 2016; for the here studied urbanization gradient: De Ridder, Maiheu, Wouters, & Lipzig, 2015; Brans et al., 2017), we focused on the extreme urban and rural populations of the gradient. Note that this approach is a relevant and powerful setting to test for the type of spatial differentiation in TPCs (horizontal vs vertical shift). To better interpret the TPC pattern for growth rate and possible costs, we also reconstructed TPCs for three other key life‐history traits: larval survival, egg development time, and hatchling body size. As study species, we chose the damselfly *Coenagrion puella*, which is very abundant in both rural and urban areas in Europe (Goertzen & Suhling, 2013). We studied rural and urban population in Flanders. At this spatial scale, populations of *C. puella* experience high gene flow (Johansson, Stoks, Nilsson‐Örtman, Ingvarsson, & Johansson, 2013), and hence, our study entails microgeographic differentiation sensu Richardson, Urban, Bolnick, and Skelly (2014). As ectothermic invertebrates, damselfly larvae are especially sensitive to temperature and have been documented to show thermal adaptation at a macrogeographic (e.g., latitudinal) scale (e.g., De Block, Pauwels, Van Den Broeck, De Meester, & Stoks, 2013; Nilsson‐Örtman, Stoks, De Block, & Johansson, 2012; Shama, Campero‐Paz, Wegner, De Block, & Stoks, 2011). Very few studies focused on effects of urbanization in damselflies (Tüzün, Debecker, Op de Beeck, & Stoks, 2015; Tüzün, Op de Beeck, & Stoks, 2017; Villalobos‐Jiménez, Dunn, & Hassall, 2016; Villalobos‐Jiménez & Hassall, 2017), and these did not consider differentiation in TPCs.

## 2 MATERIALS AND METHODS

### 2.1 Study species and populations

**Figure 1:**
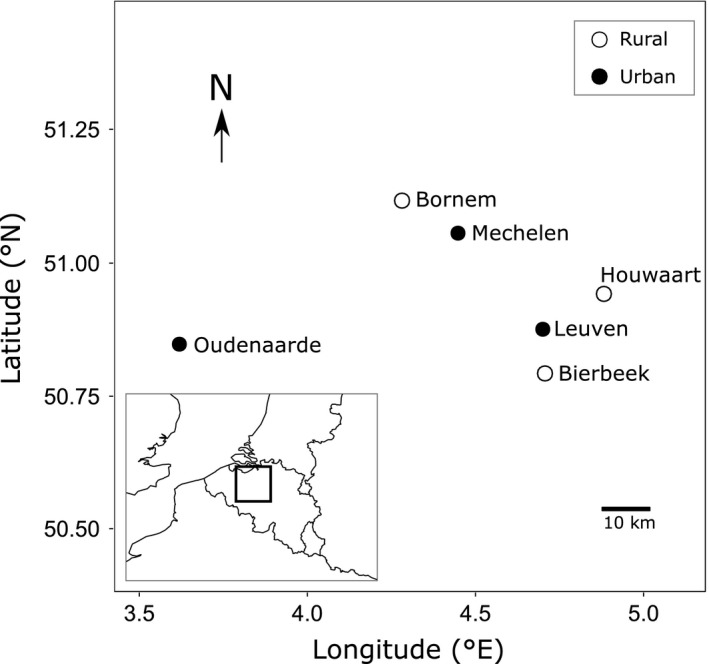
Location of the six study ponds. All ponds were located in Flanders, Belgium (inset)

Adult *C. puella* reproduce in early summer and eggs hatch ca. 3 weeks later. Larval development takes ca. 10 months (Lowe, Harvey, Watts, & Thompson, 2009). This species is univoltine in central Europe (Corbet, Suhling, & Soendgerath, 2006). We studied three rural populations (Bierbeek, Bornem, and Houwaart) and three urban populations (Leuven, Mechelen, and Oudenaarde), all situated within a 45 km radius in Flanders, Belgium (Figure 1, Appendix S1). All ponds are shallow water bodies with abundant aquatic vegetation. The selection of urban and rural ponds was carried out following a two‐step procedure using geographic information system (GIS). First, three urban and three rural 3 × 3 km plots were selected based on the percentage of built‐up area: >15% for urban plots and <3% for rural plots. Second, we selected in each plot a pond in a subplot of 200 × 200 m with the same urbanization level. This ensured that both the direct environment (subplot) and the broader surroundings (plot) reflected the same urbanization level. This sampling design was applied in several recent studies where effects of urbanization on organisms are investigated (Brans et al., 2017; Kaiser, Merckx, & Van Dyck, 2016; Piano et al., 2017; Tüzün et al., 2015, 2017). We collected eggs from 10 mated females from each of six ponds in July 2013.

### 2.2 Experimental setup

We set up a full factorial common garden experiment with five rearing temperatures crossed with two levels of urbanization (urban and rural, each represented by three populations). Animals were reared from the egg stage at one of five constant temperatures: 16, 20, 24, 28, and 30°C. Mean water temperatures experienced by the study species are typically in the range 16–20°C during the early and intermediate periods of development in summer and early fall (Nilsson‐Örtman, Stoks, De Block, & Johansson, 2013). Eggs and larvae may experience higher water temperatures during summer (R. Stoks, unpublished data). Simulations with the lake model Flake (Lake Model Flake 2016) confirmed the occurrence of the here used extreme temperatures in the study region during summer. Although we did not use the more realistic daily fluctuating temperature regime, the ranking of trait values among mean temperature treatments has been shown to be largely unaffected by fluctuating vs. constant temperature regimes (e.g., Fischer, Kölzow, Höltje, & Karl, 2011). We assigned 30 individuals (three larvae per female) per population to each temperature (total of 900 individuals).

Eggs and larvae were kept in dechlorinated tap water and placed in incubators at a photoperiod of 14:10‐h light/dark (reflecting the late summer – early fall photoperiod at the study region) at one of the five rearing temperatures. Larvae were reared individually in 200‐ml plastic cups and were fed *Artemia* nauplii 5 days a week (mean ± *SE*: 212 ± 67 nauplii per feeding portion, *n *=* *12 feeding portions), corresponding to high food levels.

To estimate growth rate, we measured the head width of each larva on days 0 (newly hatched), 30, and 50 using a digital camera attached to a binocular microscope. Head width is an often used measure to estimate size and growth in damselfly larvae (for an example in the study species: Mikolajewski, Brodin, Johansson, & Joop, 2005). The repeated measurements of head width allowed testing for ontogenetic changes in the thermal growth curves (Nilsson‐Örtman et al., 2013). As the thermal sensitivity of growth rates in the study species strongly depends on the ontogenetic stage (Nilsson‐Örtman et al., 2013; Van Doorslaer & Stoks, 2005), we calculated growth rate separately for the periods between days 0–30 and days 30–50 (from here on referred to as first and second period, respectively). This period spans the important growth period of *C. puella* larvae during late summer and early fall. To calculate growth rate, we used the formula [ln(final head size) – ln(initial head size)]/number of days. In animals experiencing seasonal time constraints, such as the here studied damselfly *C. puella* (Lowe et al., 2009; Mikolajewski, De Block, & Stoks, 2015), growth rate is a relevant performance trait. Moreover, rapid larval growth is important to reach a size advantage in cannibalistic interactions in this study species (Rolff, 1999), especially immediately following hatching when the densities are high.

We calculated larval survival as the ratio of larvae that survived up to day 50. Details on egg size, egg development time, and larval size at hatching are reported and discussed in Appendix S2. The experiment was terminated at day 50, when larvae were old enough to weigh them without causing damage (see Appendix S3 for details**)**.

### 2.3 Statistical analyses

Unless stated otherwise, all analyses were conducted with R version 3.2.2 for Windows (R Core Team 2015). We used the package “lme4” (Bates, Maechler, Bolker, & Walker, 2015) for mixed‐effects models, and the package “car” to compute Wald χ^2^ statistic and *p*‐values for fixed effects (Fox & Weisberg, 2011). Significant interactions were further analyzed by comparing least‐square means using contrast analysis.

To assess the effects of urbanization level and rearing temperature on growth rate and survival, we used separate (generalized) linear mixed‐effects models. We tested for the effects of urbanization level (urban and rural) and temperature (both linear and quadratic term) by including these terms, and their interactions, as fixed effects. As an exception, growth period (first and second period) was included as an additional fixed effect to the growth rate model. We included hatchling size as a covariate to the growth rate model. The following random effects were added where appropriate: population, nested within urbanization level (accounting that animals from the same pond are not independent replicates, thereby avoiding pseudoreplication), female identity of the offspring (accounting for among‐brood variation), individual identity of larvae (there were two growth rate estimates, one per period, for each larva; i.e., repeated‐measures design). We provide a detailed summary of the model structures in Appendix S4.

To decompose the variation in TPCs into contributions of “hotter–colder”, “faster–slower” and “generalist–specialist” models, we used the Template Mode of Variation (TMV; Izem & Kingsolver, 2005) using the code by Izem and Kingsolver (2005) implemented for Matlab (v.8.6.0). This method uses a polynomial function for the decomposition, where each direction of variation is represented by changes in curve‐specific parameters (i.e., height, width and optimum temperature). Based on the detected urban–rural differentiation in TPCs using linear mixed‐effects models (see Results), we applied this method on growth rates during the second period.

## 3 RESULTS

### 3.1 Larval growth rates

**Figure 2:**
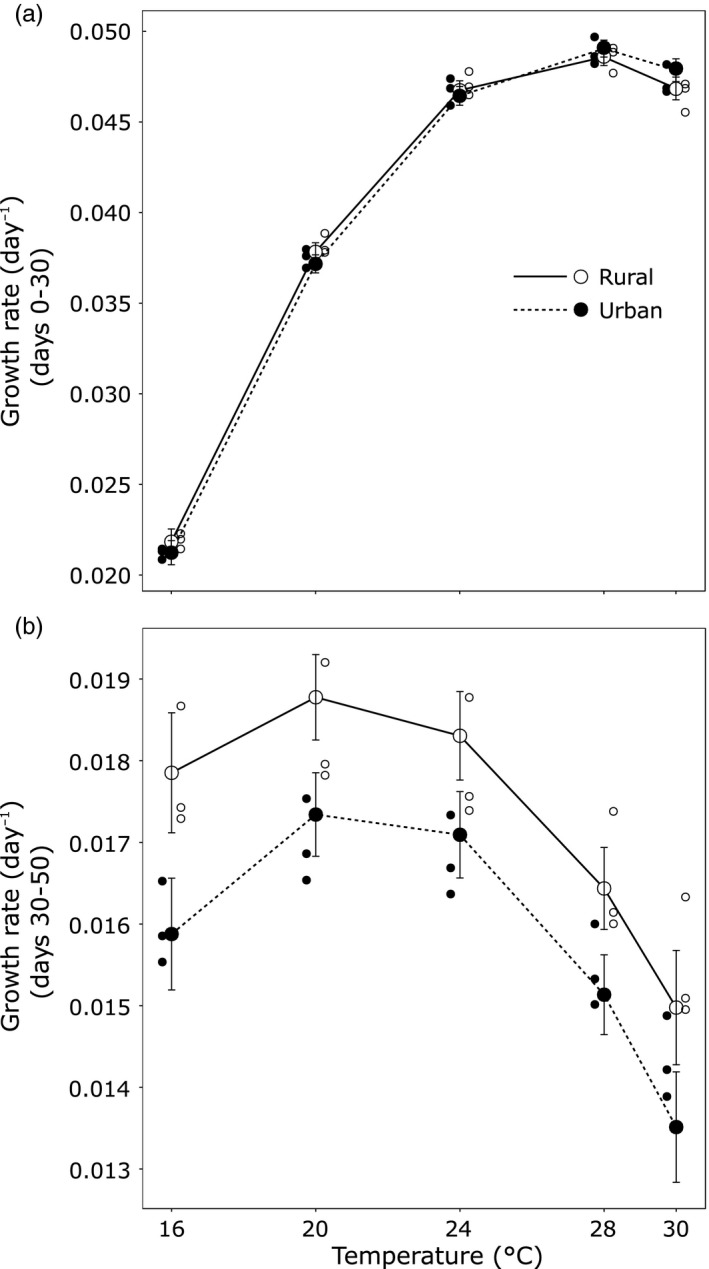
Thermal performance curves for larval growth rate between (a) days 0–30 and (b) days 30–50 of urban and rural populations of the damselfly *Coenagrion puella*. Note the different scales of the vertical axes. Small circles represent means of individual populations of a given urbanization level. Least‐square means ± 1 *SE* are shown

Larvae with smaller hatchling size had higher growth rates (χ^2^ = 20.9, *df* = 1, *p *<* *.001). Both the linear (Temperature × Growth period: χ^2^ = 225.2, *df* = 1, *p *<* *.001) and the quadratic effect of temperature on growth rate (Temperature² × Growth period: χ^2^ = 142.2, *df* = 1, *p *<* *.001) differed between the two growth periods. In addition, growth rates were ca. 2.5 times higher during the first period (0.041 day^−1^) compared to the second period (0.017 day^−1^) (χ^2^ = 8176, *df* = 1, *p *<* *.001). During the first period, increasing temperatures resulted in an increase in growth rate up to 28°C where a plateau was reached (Figure 2a). During the second period, the TPC had a concave downward shape with decreasing growth rates at temperatures above 24°C (Figure 2b).

Growth rates were higher in rural larvae than in urban larvae, but only in the second period (Urbanization level × Growth period: χ^2^ = 6.72, *df* = 1, *p = *.01; contrast test, first period: *p = *.97; second period: *p = *.02, Figure 2b). The effect of temperature on growth rate was similar for rural and urban larvae, and this was consistent across growth periods (all interactions with temperature *p > *.4).

The TMV analysis explained 64.65% of the total variation in TPCs. In line with the results from the linear mixed‐effects model, the TMV analysis of the growth rate during the second period revealed that the vertical shift (“faster–slower” model) explained the majority of the variation (58.82%) in TPCs of urban and rural larvae, whereas the horizontal shift of the TPCs (“hotter–colder” model) and the “generalist–specialist” model accounted only for 3.76% and 2.07% of the variation, respectively. Additional outputs of the TMV analysis are reported in Appendix S5.

### 3.2 Larval survival

**Figure 3:**
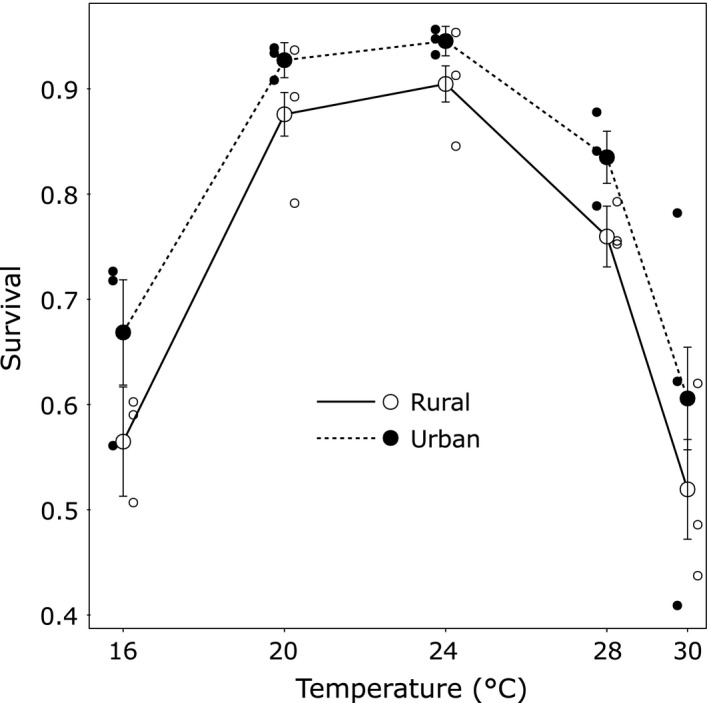
Thermal performance curves for larval survival of urban and rural populations of the damselfly *Coenagrion puella*. Small circles represent means of individual populations of a given urbanization level. Least‐square means±1 *SE* are shown

Larval survival was ca. 90% at intermediate temperatures and decreased to ca. 60% both at the lowest (16°C) and the highest (30°C) temperature, resulting in an inverted U‐shaped TPC (Figure 3); a pattern supported by the highly significant quadratic effect of temperature (χ^2^ = 122.3, *df* = 1, *p *<* *.001). Urban larvae had higher survival compared to rural larvae (χ^2^ = 5.43, *df* = 1, *p = *.02), and this was consistent along the temperature range (Urbanization level × Temperature: χ^2^ = 0.38, *df* = 1, *p = *.53; Urbanization level × Temperature²: χ^2^ = 0.41, *df* = 1, *p = *.52).

## 4 DISCUSSION

We found solid support for microgeographic differentiation of the TPCs for growth rate between urban and rural populations. Average surface temperatures during summer have been reported to be between 1.4 and 4.5°C, and in extreme cases up to 8°C, warmer in urban compared to rural areas in Flanders (De Ridder et al., 2015). In line with this, a recent study reported a 4.03°C difference in mean summer maximum water temperature in a subset of two urban and two rural ponds used in this study (Brans et al., 2017). Despite the higher water temperatures in urban ponds, the TPCs for growth rate were not shifted horizontally toward higher optima in urban damselfly populations. Furthermore, although daily temperature fluctuations are more pronounced in urban than in rural ponds in the study region (J. Engelen and K. Brans, unpublished data), there was no differentiation in the width of the TPCs. Instead, TPCs were shifted vertically with rural larvae having higher growth rates across rearing temperatures during the second period. The observation that the vertical shift in growth TPCs was consistent across the three sampled populations per urbanization level suggests this pattern to be general (see Appendix S6 and Appendix S7 for more detail). While the here chosen design of replicated populations studied at the extreme ends of the urbanization gradient is powerful to detect microgeographic shifts in TPCs, our setup does not allow to infer that these shifts occur gradually across the urbanization gradient. As we will argue that time constraints associated with changes in temperature likely are driving the vertical shift in TPCs, and given that temperatures gradually change along urbanization gradients (Parris, 2016; for the here studied urbanization gradient: De Ridder et al., 2015; Brans et al., 2017), gradual changes in TPCs are expected. This will, however, require further studies also including populations sampled at intermediate positions along the urbanization gradient.

There was a pronounced ontogenetic shift in the TPC for growth rate. While during the first period, growth rates were faster and the TPC had a steep slope and a high thermal optimum, there was a shift in the second period toward lower growth rates and a downward concave‐shaped TPC with a lower thermal optimum. Such ontogenetic shifts in TPC for growth have been associated with the decreasing temperatures larvae are exposed to during early and intermediate growth periods, and have been reported before in *Coenagrion* damselfly species, including the study species *C. puella* (Nilsson‐Örtman et al., 2013; Van Doorslaer & Stoks, 2005). The high thermal optimum in the first period (28°C) is not often reached in the study ponds. This fits the pattern that thermal optima are typically higher than modal environmental temperatures (Angilletta, Huey, & Frazier, 2010). This has been explained by the typically asymmetric shape of TPCs resulting in drastically lower performance above the thermal optimum than below the thermal optimum (Deutsch et al., 2008). Even if the optimal temperatures are met infrequently, it may still be beneficial to optimize growth at higher temperatures as even short exposures to higher temperatures may allow significant gains (Kingsolver, 2000). Selection for high growth rates and reaching a larger size may be stronger in early larval stages because of the initial high larval densities (leading to increased cannibalism; Hopper, Crowley, & Kielman, 1996) and higher temperatures (leading to increased competition and intraguild predation; Brown, Gillooly, Allen, Savage, & West, 2004). This, together with the identified trade‐off between early and late growth rates in *Coenagrion* damselfly larvae (Nilsson‐Örtman et al., 2013), may have caused the higher growth rates in the first compared to the second period. Alternatively, the lower growth rates in the second period could be an artifact driven by size‐dependent growth rate (Tammaru & Esperk, 2007), where the lower growth rates of the larvae during the second period were due to their on average larger size. This scenario, however, is unlikely to drive the observed pattern as we controlled for the larval size (by including either initial size or time‐varying sizes as covariate; see Appendix S3) when analyzing growth rate. Note that given size is fixed per developmental stage, this also corrects for any differences in developmental stage between temperature treatments. Although we followed the growth rate TPCs only until day 50, this captures the major part of the prewinter growth period. Moreover, for the study species, the shape of the TPCs between days 30 and 50 reflects the TPCs until the end of the prewinter growing season (based on Van Doorslaer & Stoks, 2005; Nilsson‐Örtman, Rogell, Stoks, & Johansson, 2015).

A key finding of our study was the vertical shift in TPC for growth rate during the second period with higher growth rates in rural larvae compared to urban larvae. This vertical shift in TPCs is consistent with a pattern of countergradient variation (Conover & Schultz, 1995; Conover et al., 2009). We did not find any evidence for the theoretically predicted higher thermal optima (“hotter–colder” model, Lynch & Gabriel, 1987; Gilchrist, 1995) of urban damselfly larvae that would suggest thermal adaptation. The majority of evidence for thermal adaptation comes from interspecific studies (e.g., Angilletta et al., 2010; Frazier, Huey, & Berrigan, 2006), whereas intraspecific studies usually favor the “faster–slower” model (e.g., Izem & Kingsolver, 2005; Richter‐Boix et al., 2015; Yamahira & Conover, 2002). It has been suggested that thermal adaptation (“hotter–colder” model) requires radical changes in the genetic structure (e.g., mutations that would allow for an increased growth via horizontal shifts in thermokinetics of enzyme function), which requires time of the magnitude similar to what is needed for the divergence of species (Yamahira & Conover, 2002; Yamahira, Kawajiri, Takeshi, & Irie, 2007). Furthermore, in spite of the higher daily temperature fluctuations in urban ponds (J. Engelen and K. Brans, unpublished data), urban populations did not have wider TPCs (“generalist–specialist” model; Lynch & Gabriel, 1987; Gilchrist, 1995). This matches the general pattern that thermal specialization is only encountered infrequently (Angilletta, 2009; but see Latimer, Wilson, & Chenoweth, 2011; Richter‐Boix et al., 2015).

The majority of studies reporting vertical shifts in TPCs for growth rates show this pattern across large geographic gradients with little gene flow among populations (Conover et al., 2009), whereas only a handful of studies demonstrated this pattern at a microgeographic scale where gene flow can be high (but see Blanckenhorn, 1991; Skelly, 2004; Richter‐Boix, Teplitsky, Rogell, & Laurila, 2010; Richter‐Boix et al., 2015). Also in the here studied *C. puella*, gene flow is high at the studied scale in Flanders (as indicated by the very low *F*_ST_‐values of ca. 0.02 for this study species, see Figure 2 in Johansson et al., 2013). This indicates that selection for higher growth rates is especially strong to counteract the homogenizing force of gene flow, thereby supporting accumulating evidence that local adaptation may occur at small spatial scales in the presence of gene flow (reviewed in Richardson et al., 2014).

Given that we obtained the TPC differentiation in a common garden experiment suggests that the pattern reflects genetic adaptation, rather than environmental differences. Although we used first generation animals, maternal effects seem unlikely to have played a major role because rural and urban populations did not differ in egg size and hatchling size (Appendix S2), while differences in egg size are the most common way how maternal effects are transferred (Mousseau & Dingle, 1991). More importantly, larval growth rates did not differ between rural and urban populations during the first period. It seems unlikely that any nongenetic effects transferred by the mother would have caused differences in growth rate between population types that would not be present during the first growth period. Indeed, if anything, maternal effects tend to decay throughout ontogeny (e.g., Lindholm, Hunt, & Brooks, 2006). This fits the pattern based on quantitative genetic rearing experiments that maternal effects on growth rate are absent or small in damselfly larvae (Shama et al., 2011; Sniegula, Golab, Drobniak, & Johansson, 2016). Nevertheless, we cannot fully exclude mechanisms acting via maternal effects such as transgenerational plasticity (e.g., Richter‐Boix, Orizaola, & Laurila, 2014).

The observation that the vertical shift in TPCs only was apparent in the second period may be explained by the stronger time constraints, the major selective force underlying a vertical shift in TPCs for growth rates (Conover et al., 2009). Indeed, during the second period, temperatures encountered in the field are lower while larvae face stronger time constraints to reach a certain developmental stage before the onset of winter. In support of a stronger selection to accelerate growth rates in rural populations, growing seasons are reported to be considerably shorter in rural than in urban areas (Yang et al., 2013; Somers et al., 2013; for the study region: J. Engelen and K. Brans, unpublished data). Higher growth rates under time constraints have been theoretically predicted (Abrams, Leimar, Nylin, & Wiklund, 1996) and empirically shown in *Coenagrion* damselfly larvae, including the study species (Mikolajewski et al., 2015). However, systematic differences between urban and rural ponds other than temperature might have contributed to the differentiation in TPCs. Notably, differences in predation pressure have been identified as a key factor driving the shift in TPCs for growth rate (Richter‐Boix, Quintela, Kierczak, Franc, & Laurila, 2013). Predators of damselfly larvae did, however, not differ in densities between the here studied urban and rural ponds (see Appendix S8). In agreement with our finding, aquatic macro‐invertebrate community structures are not driven by urbanization in the study region (C. Souffreau, personal communication), and a recent study reported no difference in species richness of aquatic macro‐invertebrates from urban and nonurban ponds in the United Kingdom (Hill et al., 2017). Nevertheless, even when predator densities did not differ between urban and rural ponds, predators may impose stronger selection for reduced growth rates in the urban ponds because of the typical higher predation rates at warmer temperatures (e.g., De Block et al., 2013; Sentis, Morisson, & Boukal, 2015). Larvae of the study species indeed reduce growth rates in the presence of predators (Mikolajewski et al., 2005). The higher temperatures in the urban populations may therefore be driving the lower growth rates in urban populations both by causing lower time constraints and by generating higher predation rates. Furthermore, differences in pollution levels between pond types might have contributed to the observed shift in TPCs. Indeed, tadpoles exposed to a herbicide had on average upwards shifted (“faster–slower” differentiation) and narrower TPCs (“generalist–specialist” differentiation) for swimming speed (Katzenberger et al., 2014). Yet, the expected higher contamination levels in urban waterbodies (e.g., Gilliom, 2007), together with the absence of a generalist–specialist trade‐off in our study, make this scenario unlikely.

Despite the widespread evidence of urban heat islands (Gaston et al., 2010), surprisingly few studies documented differentiation in TPCs between rural and urban populations, and as these all directly measured traits on field‐collected animals, it is unknown to what extent these differentiations are genetic rather than environmentally driven (reviewed in Chown & Duffy, 2015). In the only study considering continuous TPCs, which is essential to discriminate between the models (Richter‐Boix et al., 2015), two different patterns of thermal differentiation were observed when rearing four species of soil fungi directly isolated from one urban population and from one rural population (McLean, Angilletta, & Williams, 2005). Two fungus species followed the “hotter–colder” model, with isolates from the urban population growing faster at 26°C, but slower at 18°C compared to isolates from the rural population. In the two other fungus species, urban isolates grew as fast or faster at all temperatures than the rural isolates (“faster–slower” model). This is the opposite vertical shift in TPCs of that we here documented. The authors suggested this is because high temperatures in urban areas inhibit fungal growth, resulting in restricted growing seasons for urban populations (McLean et al., 2005), while the higher temperatures in the urban damselfly populations, instead, were beneficial and increased the length of the growing season. In contrast, two studies report higher heat tolerance in urban compared to rural populations of field‐collected leafcutter ants (Angilletta et al., 2007) and common garden‐reared acorn ants (Diamond, Chick, Perez, Strickler, & Martin, 2017), yet none of them tested differential thermal responses in life history. Finally, Brans et al. (2017) have shown genetic adaptation to urbanization in terms of a higher heat tolerance in urban populations of the water flea *Daphnia magna*.

Studies on vertical shifts in TPCs typically assume life‐history trade‐offs where the faster growing genotypes pay costs that preclude them from occupying the entire thermal gradient (Conover & Schultz, 1995; Conover et al., 2009). Costs of rapid growth may be manifold (Dmitriew, 2011), and in damselfly larvae include decreased investment in energy storage (Stoks, De Block, & McPeek, 2006), reduced immune function (De Block & Stoks, 2008b), and increased oxidative stress (De Block & Stoks, 2008a). These costs may explain the overall lower survival in rural compared to urban populations. Similarly, Hong and Shurin (2015) showed that northern populations of the tidepool copepod *Tigriopus californicus* grew faster than southern populations, yet suffered a reduced survival. However, we cannot exclude the possibility that adaptations to urbanization‐related stressors (e.g., pollution) might have resulted in an overall higher resilience of urban populations, reflected in their higher survival.

Urbanization is a major driver of microevolutionary change (Alberti, 2015; Alberti et al., 2017). Urban environments have the potential to provide unique information and aid in developing predictions on the impact of climate change on organisms (Chown & Duffy, 2015; Youngsteadt et al., 2015, 2017), and the use of cities as natural experiments has recently been promoted. Our finding that individuals from urban and rural populations consistently differed in growth rate and survival is relevant for predicting climate change impact, as the summer temperature difference between urban and rural ponds falls within the range of the expected temperature increases by 2100 under several IPCC (2013) scenarios. The urban populations can therefore be used as proxies to understand and predict the impact of global warming in rural populations under gradual evolution (Stoks, Geerts, & De Meester, 2014). Aside from contributing to the limited evidence for vertical shifts in TPCs at a microgeographic scale (Richter‐Boix et al., 2015; Skelly, 2004), this study is the first using replicated populations in a common garden experiment from the egg stage to report evidence for urbanization‐associated countergradient variation in a key performance trait.

## DATA ARCHIVING STATEMENT

Data available from the Dryad Digital Repository: https://doi.org/10.5061/dryad.9f94k

## References

[eva12512-bib-0001] Abrams, P. A. , Leimar, O. , Nylin, S. , & Wiklund, C. (1996). The effect of flexible growth rates on optimal sizes and development times in a seasonal environment. American Naturalist, 147, 381–395.

[eva12512-bib-0002] Alberti, M. (2015). Eco‐evolutionary dynamics in an urbanizing planet. Trends in Ecology and Evolution, 30, 1–13. 25498964 10.1016/j.tree.2014.11.007

[eva12512-bib-0003] Alberti, M. , Correa, C. , Marzluff, J. M. , Hendry, A. P. , Palkovacs, E. P. , Gotanda, K. M. , … Zhou, Y. (2017). Global urban signatures of phenotypic change in animal and plant populations. Proceedings of the National Academy of Sciences of the United States of America. https://doi.org/10.1073/pnas.1606034114 10.1073/pnas.1606034114 PMC5576774 28049817

[eva12512-bib-0004] Angilletta, M. J. (2009). Thermal adaptation: A theoretical and empirical synthesis. New York: Oxford University Press.

[eva12512-bib-0005] Angilletta, M. J. , Huey, R. B. , & Frazier, M. R. (2010). Thermodynamic effects on organismal performance: Is hotter better? Physiological and Biochemical Zoology, 83, 197–206. 20001251 10.1086/648567

[eva12512-bib-0006] Angilletta, M. J. , Wilson, R. S. , Niehaus, A. C. , Sears, M. W. , Navas, C. A. , & Ribeiro, P. L. (2007). Urban physiology: City ants possess high heat tolerance. PLoS ONE, 2, e258. 17327918 10.1371/journal.pone.0000258 PMC1797824

[eva12512-bib-0007] Bates, D. , Maechler, M. , Bolker, B. , & Walker, S. (2015). Fitting linear mixed‐effects models using lme4. Journal of Statistical Software, 67, 1–48.

[eva12512-bib-0008] Blanckenhorn, W. U. (1991). Life‐history difference in adjacent water strider populations: Phenotypic plasticity or heritable responses to stream temperature? Evolution, 45, 1520–1525. 28563826 10.1111/j.1558-5646.1991.tb02655.x

[eva12512-bib-0009] Brans, K. I. , Govaert, L. , Engelen, J. , Gianuca, A. T. , Souffreau, C. , & De Meester, L. (2017). Eco‐evolutionary dynamics in urbanized landscapes: Evolution, species sorting and the change in zooplankton body size along urbanization gradients. Philosophical transactions of the Royal Society of London. Series B, Biological sciences, 372, 20160030. 27920375 10.1098/rstb.2016.0030 PMC5182426

[eva12512-bib-0010] Brans, K. I. , Jansen, M. , Vanoverbeke, J. , Tüzün, N. , Stoks, R. , & De Meester, L . (2017). The heat is on: Genetic adaptation to urbanization mediated by thermal tolerance and body size. Global Change Biology. https://doi.org/10.1111/gcb.13784 10.1111/gcb.13784 28614592

[eva12512-bib-0011] Brown, J. H. , Gillooly, J. F. , Allen, A. P. , Savage, V. M. , & West, G. B. (2004). Toward a metabolic theory of ecology. Ecology, 85, 1771–1789.

[eva12512-bib-0012] Chown, S. L. , & Duffy, G. A. (2015). Thermal physiology and urbanization: Perspectives on exit, entry and transformation rules. Functional Ecology, 29, 902–912.

[eva12512-bib-0013] Conover, D. O. , Duffy, T. A. , & Hice, L. A. (2009). The covariance between genetic and environmental influences across ecological gradients. Annals of the New York Academy of Sciences, 1168, 100–129. 19566705 10.1111/j.1749-6632.2009.04575.x

[eva12512-bib-0014] Conover, D. O. , & Schultz, E. T. (1995). Phenotypic similarity and the evolutionary significance of countergradient variation. Trends in Ecology and Evolution, 10, 248–252. 21237029 10.1016/S0169-5347(00)89081-3

[eva12512-bib-0015] Corbet, P. , Suhling, F. , & Soendgerath, D. (2006). Voltinism of Odonata: A review. International Journal of Odonatology, 9, 1–44.

[eva12512-bib-0016] De Block, M. , Pauwels, K. , Van Den Broeck, M. , De Meester, L. , & Stoks, R. (2013). Local genetic adaptation generates latitude‐specific effects of warming on predator‐prey interactions. Global Change Biology, 19, 689–696. 23504827 10.1111/gcb.12089

[eva12512-bib-0017] De Block, M. , & Stoks, R. (2008a). Compensatory growth and oxidative stress in a damselfly. Proceedings of the Royal Society B, 275, 781–785. 18182373 10.1098/rspb.2007.1515 PMC2596904

[eva12512-bib-0018] De Block, M. , & Stoks, R. (2008b). Short‐term larval food stress and associated compensatory growth reduce adult immune function in a damselfly. Ecological Entomology, 33, 796–801.

[eva12512-bib-0019] De Ridder, K. , Maiheu, B. , Wouters, H. , & vanLipzig, N . (2015). Indicatoren van het stedelijk hitte‐eiland in Vlaanderen, studie uitgevoerd in opdracht van de Vlaamse Milieumaatschappij MIRA/2015/05, VITO. http://www.milieurapport.be/Upload/main/0_Klimaatrapport/2015-05_MIRA_UHI_eindrapport_ TW2.pdf.

[eva12512-bib-0020] Deutsch, C. A. , Tewksbury, J. J. , Huey, R. B. , Sheldon, K. S. , Ghalambor, C. K. , Haak, D. C. , & Martin, P. R. (2008). Impacts of climate warming on terrestrial ectotherms across latitude. Proceedings of the National Academy of Sciences of the United States of America, 105, 6668–6672. 18458348 10.1073/pnas.0709472105 PMC2373333

[eva12512-bib-0021] Diamond, S. E. , Chick, L. , Perez, A. , Strickler, S. A. , & Martin, R. A. (2017). Rapid evolution of ant thermal tolerance across an urban‐rural temperature cline. Biological Journal of the Linnean Society, https://doi.org/10.1093/biolinnean/blw047

[eva12512-bib-0022] Diamond, S. E. , Dunn, R. R. , Frank, S. D. , Haddad, N. M. , & Martin, R. A. (2015). Shared and unique responses of insects to the interaction of urbanization and background climate. Current Opinion in Insect Science, 11, 71–77. 28285761 10.1016/j.cois.2015.10.001

[eva12512-bib-0023] Dmitriew, C. M. (2011). The evolution of growth trajectories: What limits growth rate? Biological Reviews, 86, 97–116. 20394607 10.1111/j.1469-185X.2010.00136.x

[eva12512-bib-0024] Fischer, K. , Kölzow, N. , Höltje, H. , & Karl, I. (2011). Assay conditions in laboratory experiments: Is the use of constant rather than fluctuating temperatures justified when investigating temperature‐induced plasticity? Oecologia, 166, 23–33. 21286923 10.1007/s00442-011-1917-0

[eva12512-bib-0025] Fox, J. , & Weisberg, S. (2011). An R companion to applied regression, 2nd ed. Thousand Oaks, California: Sage.

[eva12512-bib-0026] Frazier, M. R. , Huey, R. B. , & Berrigan, D. (2006). Thermodynamics constrains the evolution of insect population growth rates: “warmer is better”. The American Naturalist, 168, 512–520. 10.1086/506977 17004222

[eva12512-bib-0027] Gaston, K. J. , Davies, Z. G. , & Edmondson, J. L . (2010). Urban environments and ecosystem functions In Gaston K. J. (Ed.), Urban ecology (pp. 35–52). New York, NY: Cambridge University Press.

[eva12512-bib-0028] Gilchrist, G. W. (1995). Specialists and generalists in changing environments. I. Fitness landscapes of thermal sensitivity. American Naturalist, 146, 252–270.

[eva12512-bib-0029] Gilliom, R. (2007). Pesticides in US streams and groundwater. Environmental Science & Technology, 41, 3408–3414. 17547156 10.1021/es072531u

[eva12512-bib-0030] Goertzen, D. , & Suhling, F. (2013). Promoting dragonfly diversity in cities: Major determinants and implications for urban pond design. Journal of Insect Conservation, 17, 399–409.

[eva12512-bib-0031] Hill, M. J. , Biggs, J. , Thornhill, I. , Briers, R. A. , Gledhill, D. G. , White, J. C. , … Hassall, C. (2017). Urban ponds as an aquatic biodiversity resource in modified landscapes. Global Change Biology, 23, 986–999. 27476680 10.1111/gcb.13401

[eva12512-bib-0032] Hong, B. C. , & Shurin, J. B. (2015). Latitudinal variation in the response of tidepool copepods to mean and daily range in temperature. Ecology, 96, 2348–2359. 26594693 10.1890/14-1695.1

[eva12512-bib-0033] Hopper, K. R. , Crowley, P. H. , & Kielman, D. (1996). Density dependence, hatching synchrony, and within‐cohort cannibalism in young dragonfly larvae. Ecology, 77, 191–200.

[eva12512-bib-0034] IPCC . (2013). Working Group I Contribution to the IPCC Fifth Assessment Report, Climate Change 2013: The Physical Science Basis (Stocker T. F., Qin D., Plattner G.‐K., Tignor M., Allen S. K., Boschung J., … Midgley P. M. eds). Cambridge University Press.

[eva12512-bib-0035] Izem, R. , & Kingsolver, J. G. (2005). Variation in continuous reaction norms: Quantifying directions of biological interest. American Naturalist, 166, 277–289. 10.1086/431314 16032579

[eva12512-bib-0036] Johansson, H. , Stoks, R. , Nilsson‐Örtman, V. , Ingvarsson, P. K. , & Johansson, F. (2013). Large‐scale patterns in genetic variation, gene flow and differentiation in five species of European Coenagrionid damselfly provide mixed support for the central‐marginal hypothesis. Ecography, 36, 744–755.

[eva12512-bib-0037] Kaiser, A. , Merckx, T. , & Van Dyck, H. (2016). The Urban Heat Island and its spatial scale dependent impact on survival and development in butterflies of different thermal sensitivity. Ecology and Evolution, 6, 4129–4140. 27516869 10.1002/ece3.2166 PMC4972237

[eva12512-bib-0038] Katzenberger, M. , Hammond, J. , Duarte, H. , Tejedo, M. , Calabuig, C. , & Relyea, R. A. (2014). Swimming with predators and pesticides: How environmental stressors affect the thermal physiology of tadpoles. PLoS ONE, 9, 1–11. 10.1371/journal.pone.0098265 PMC4037208 24869960

[eva12512-bib-0039] Kingsolver, J. G. (2000). Feeding, growth, and the thermal environment of cabbage white caterpillars, *Pieris rapae* L. Physiological and Biochemical Zoology, 73, 621–628. 11073798 10.1086/317758

[eva12512-bib-0040] Lake Model Flake (2016). Flake online. Retrieved from http://www.flake.igb-berlin.de/

[eva12512-bib-0041] Latimer, C. A. L. , Wilson, R. S. , & Chenoweth, S. F. (2011). Quantitative genetic variation for thermal performance curves within and among natural populations of *Drosophila serrata* . Journal of Evolutionary Biology, 24, 965–975. 21306462 10.1111/j.1420-9101.2011.02227.x

[eva12512-bib-0042] Lindholm, A. K. , Hunt, J. , & Brooks, R. (2006). Where do all the maternal effects go? Variation in offspring body size through ontogeny in the live‐bearing fish *Poecilia parae* . Biology Letters, 2, 586–589. 17148295 10.1098/rsbl.2006.0546 PMC1833979

[eva12512-bib-0043] Lowe, C. D. , Harvey, I. F. , Watts, P. C. , & Thompson, D. J. (2009). Reproductive timing and patterns of development for the damselfly *Coenagrion puella* in the field. Ecology, 90, 2202–2212. 19739382 10.1890/08-1780.1

[eva12512-bib-0044] Lynch, M. , & Gabriel, W. (1987). Environmental tolerance. American Naturalist, 129, 283–303. 10.1086/432558 16224689

[eva12512-bib-0045] McLean, M. A. , Angilletta, M. J. , & Williams, K. S. (2005). If you can't stand the heat, stay out of the city: Thermal reaction norms of chitinolytic fungi in an urban heat island. Journal of Thermal Biology, 30, 384–391.

[eva12512-bib-0046] Mikolajewski, D. J. , Brodin, T. , Johansson, F. , & Joop, G. (2005). Phenotypic plasticity in gender specific life‐history: Effects of food availability and predation. Oikos, 110, 91–100.

[eva12512-bib-0047] Mikolajewski, D. J. , De Block, M. , & Stoks, R. (2015). The interplay of adult and larval time constraints shapes species differences in larval life history. Ecology, 96, 1128–1138. 26230032 10.1890/14-0262.1

[eva12512-bib-0048] Mousseau, T. A. , & Dingle, H . (1991). Maternal effects in insect life histories. Annual Review of Entomology, 36, 511–534.

[eva12512-bib-0049] Neil, K. , & Wu, J. (2006). Effects of urbanization on plant flowering phenology: A review. Urban Ecosystems, 9, 243–257.

[eva12512-bib-0050] Nilsson‐Örtman, V. , Rogell, B. , Stoks, R. , & Johansson, F. (2015). Ontogenetic changes in genetic variances of age‐dependent plasticity along a latitudinal gradient. Heredity, 115, 366–378. 25649500 10.1038/hdy.2014.126 PMC4815462

[eva12512-bib-0051] Nilsson‐Örtman, V. , Stoks, R. , De Block, M. , & Johansson, F. (2012). Generalists and specialists along a latitudinal transect: Patterns of thermal adaptation in six species of damselflies. Ecology, 93, 1340–1352. 22834375 10.1890/11-1910.1

[eva12512-bib-0052] Nilsson‐Örtman, V. , Stoks, R. , De Block, M. , & Johansson, F. (2013). Latitudinal patterns of phenology and age‐specific thermal performance across six *Coenagrion* damselfly species. Ecological Monographs, 83, 491–510.

[eva12512-bib-0053] Parris, K. M . (2016). Urban environments In Parris K. M. (Ed.), Ecology of urban environments (pp. 15–41). Chichester, UK: Wiley‐Blackwell Publishing.

[eva12512-bib-0054] Piano, E. , De Wolf, K. , Bona, F. , Bonte, D. , Bowler, D. E. , Isaia, M. , … Hendrickx, F. (2017). Urbanization drives community shifts towards thermophilic and dispersive species at local and landscape scales. Global Change Biology, https://doi.org/10.1111/gcb.13606 10.1111/gcb.13606 27997069

[eva12512-bib-0055] R Core Team . (2015). R: A language and environment for statistical computing. Vienna, Austria: R Foundation for Statistical Computing.

[eva12512-bib-0056] Richardson, J. L. , Urban, M. C. , Bolnick, D. I. , & Skelly, D. K. (2014). Microgeographic adaptation and the spatial scale of evolution. Trends in Ecology and Evolution, 29, 165–176. 24560373 10.1016/j.tree.2014.01.002

[eva12512-bib-0057] Richter‐Boix, A. , Katzenberger, M. , Duarte, H. , Quintela, M. , Tejedo, M. , & Laurila, A. (2015). Local divergence of thermal reaction norms among amphibian populations is affected by pond temperature variation. Evolution, 69, 2210–2226. 26118477 10.1111/evo.12711

[eva12512-bib-0058] Richter‐Boix, A. , Orizaola, G. , & Laurila, A. (2014). Transgenerational phenotypic plasticity links breeding phenology with offspring life‐history. Ecology, 95, 2715–2722.

[eva12512-bib-0059] Richter‐Boix, A. , Quintela, M. , Kierczak, M. , Franc, M. , & Laurila, A. (2013). Fine‐grained adaptive divergence in an amphibian: Genetic basis of phenotypic divergence and the role of non‐random gene flow in restricting effective migration among wetlands. Molecular Ecology, 22, 1322–1340. 23294180 10.1111/mec.12181

[eva12512-bib-0060] Richter‐Boix, A. , Teplitsky, C. , Rogell, B. , & Laurila, A. (2010). Local selection modifies phenotypic divergence among *Rana temporaria* populations in the presence of gene flow. Molecular Ecology, 19, 716–731. 20089126 10.1111/j.1365-294X.2009.04502.x

[eva12512-bib-0061] Rolff, J. (1999). Parasitism increases offspring size in a damselfly: Experimental evidence for parasite‐mediated maternal effects. Animal Behaviour, 58, 1105–1108. 10564613 10.1006/anbe.1999.1240

[eva12512-bib-0062] Sentis, A. , Morisson, J. , & Boukal, D. S. (2015). Thermal acclimation modulates the impacts of temperature and enrichment on trophic interaction strengths and population dynamics. Global Change Biology, 21, 3290–3298. 25808556 10.1111/gcb.12931

[eva12512-bib-0063] Seto, K. C. , Güneralp, B. , & Hutyra, L. R. (2012). Global forecasts of urban expansion to 2030 and direct impacts on biodiversity and carbon pools. Proceedings of the National Academy of Sciences of the United States of America, 109, 16083–16088. 22988086 10.1073/pnas.1211658109 PMC3479537

[eva12512-bib-0064] Shama, L. N. S. , Campero‐Paz, M. , Wegner, K. M. , De Block, M. , & Stoks, R. (2011). Latitudinal and voltinism compensation shape thermal reaction norms for growth rate. Molecular Ecology, 20, 2929–2941. 21689189 10.1111/j.1365-294X.2011.05156.x

[eva12512-bib-0065] Sinclair, B. J. , Marshall, K. E. , Sewell, M. A. , Levesque, D. L. , Willett, C. S. , Slotsbo, S. , … Huey, R. B. (2016). Can we predict ectotherm responses to climate change using thermal performance curves and body temperatures? Ecology Letters, 19, 1372–1385. 27667778 10.1111/ele.12686

[eva12512-bib-0066] Skelly, D. K. (2004). Microgeographic countergradient variation in the wood frog, *Rana sylvatica* . Evolution, 58, 160–165. 15058728 10.1111/j.0014-3820.2004.tb01582.x

[eva12512-bib-0067] Sniegula, S. , Golab, M. J. , Drobniak, S. M. , & Johansson, F. (2016). Seasonal time constraints reduce genetic variation in life‐history traits along a latitudinal gradient. Journal of Animal Ecology, 85, 187–198. 26333659 10.1111/1365-2656.12442

[eva12512-bib-0068] Somers, K. A. , Bernhardt, E. S. , Grace, J. B. , Hassett, B. A. , Sudduth, E. B. , Wang, S. , & Urban, D. L. (2013). Streams in the urban heat island: Spatial and temporal variability in temperature. Freshwater Science, 32, 309–326.

[eva12512-bib-0069] Stinchcombe, J. R. , Function‐Valued Traits Working Group , & Kirkpatrick, M. (2012). Genetics and evolution of function‐valued traits: Understanding environmentally responsive phenotypes. Trends in Ecology and Evolution, 27, 637–647. 22898151 10.1016/j.tree.2012.07.002

[eva12512-bib-0070] Stoks, R. , De Block, M. , & McPeek, M. A. (2006). Physiological costs of compensatory growth in a damselfly. Ecology, 87, 1566–1574. 16869432 10.1890/0012-9658(2006)87[1566:pcocgi]2.0.co;2

[eva12512-bib-0071] Stoks, R. , Geerts, A. N. , & De Meester, L. (2014). Evolutionary and plastic responses of freshwater invertebrates to climate change: Realized patterns and future potential. Evolutionary Applications, 7, 42–55. 24454547 10.1111/eva.12108 PMC3894897

[eva12512-bib-0072] Tammaru, T. , & Esperk, T. (2007). Growth allometry of immature insects: Larvae do not grow exponentially. Functional Ecology, 21, 1099–1105.

[eva12512-bib-0073] Townroe, S. , & Callaghan, A. (2014). British container breeding mosquitoes: The impact of urbanisation and climate change on community composition and chenology. PLoS ONE, 9, e95325. 24759617 10.1371/journal.pone.0095325 PMC3997353

[eva12512-bib-0074] Tüzün, N. , Debecker, S. , Op de Beeck, L. , & Stoks, R. (2015). Urbanisation shapes behavioural responses to a pesticide. Aquatic Toxicology, 163, 81–88. 25863029 10.1016/j.aquatox.2015.04.002

[eva12512-bib-0075] Tüzün, N. , Op de Beeck, L. , & Stoks, R. (2017). Sexual selection reinforces a higher flight endurance in urban damselflies. Evolutionary Applications, 10, 694–703. 28717389 10.1111/eva.12485 PMC5511363

[eva12512-bib-0076] Van Doorslaer, W. , & Stoks, R. (2005). Thermal reaction norms in two *Coenagrion* damselfly species: Contrasting embryonic and larval life‐history traits. Freshwater Biology, 50, 1982–1990.

[eva12512-bib-0077] Villalobos‐Jiménez, G. , Dunn, A. M. , & Hassall, C. (2016). Dragonflies and damselflies (Odonata) in urban ecosystems: A review. European Journal of Entomology, 113, 217–232.

[eva12512-bib-0078] Villalobos‐Jiménez, G. , & Hassall, C. (2017). Effects of the urban heat island on the phenology of Odonata in London, UK. International Journal of Biometeorology, 61, 1337–1346. 28190181 10.1007/s00484-017-1311-7 PMC5486733

[eva12512-bib-0079] Yamahira, K. , & Conover, D. O. (2002). Intra‐ vs. inter‐ specific latitudinal variation in growth: Adaptation to temperature or seasonality? Ecology, 83, 1252–1262.

[eva12512-bib-0080] Yamahira, K. , Kawajiri, M. , Takeshi, K. , & Irie, T. (2007). Inter‐ and intrapopulation variation in thermal reaction norms for growth rate: Evolution of latitudinal compensation in ectotherms with a genetic constraint. Evolution, 61, 1577–1589. 17598741 10.1111/j.1558-5646.2007.00130.x

[eva12512-bib-0081] Yang, X. , Tian, Z. , & Chen, B. (2013). Thermal growing season trends in east China, with emphasis on urbanization effects. International Journal of Climatology, 33, 2402–2412.

[eva12512-bib-0082] Youngsteadt, E. , Dale, A. G. , Terando, A. J. , Dunn, R. R. , & Frank, S. D. (2015). Do cities simulate climate change? A comparison of herbivore response to urban and global warming. Global Change Biology, 21, 97–105. 25163424 10.1111/gcb.12692

[eva12512-bib-0083] Youngsteadt, E. , Ernst, A. F. , Dunn, R. R. , & Frank, S. D. (2017). Responses of arthropod populations to warming depend on latitude: Evidence from urban heat islands. Global Change Biology, 23, 1436–1447. 27809387 10.1111/gcb.13550

[eva12512-bib-0084] Zipper, S. C. , Schatz, J. , Singh, A. , Kucharik, C. J. , Townsend, P. A. , & Loheide, S. P. (2016). Urban heat island impacts on plant phenology: Intra‐urban variability and response to land cover. Environmental Research Letters, 11, 54023.

