# Supplementary material for: Microgeographic differentiation in thermal performance curves between rural and urban populations of an aquatic insect
Source: Evol Appl. 2017 Aug 2;10(10):1067–75. doi: 10.1111/eva.12512 (PMC5680628; doi:10.1111/eva.12512)
Supplement: Supplementary file 1 [file EVA-10-1067-s001.docx]

**Supplementary Information**

Microgeographic differentiation in thermal performance curves between rural and urban populations of an aquatic insect

**S1 –** Study populations

**S2 –** TPCs for egg development time and size at hatching

**S3 –** Complementary growth rate analyses

**S4 –** Structure of mixed-effect models

**S5** – TMV parameters

**S6 –** Testing for the consistency of populations within a given urbanization level

**S7 –** Assessing the influence of random effects

**S8 –** Predator communities

**S1 – Study populations**

**Table S1.** Coordinates and pairwise Euclidean geographic distances (in km) between the study ponds. Pond codes are given in brackets.

|  | Rural populations | | | Urban populations | | |
| --- | --- | --- | --- | --- | --- | --- |
|  | Bierbeek (BIE) | Bornem (BOR) | Houwaart (HOU) | Leuven (LEU) | Mechelen (MEC) | Oudenaarde (OUD) |
| Bierbeek | 50°47'27.87"N  4°42'12.17"E |  |  |  |  |  |
| Bornem | 46.75 | 51°6'54.40"N  4°16'41.4"E |  |  |  |  |
| Houwaart | 20.67 | 46.27 | 50°56'25.75"N  4°52'43.11"E |  |  |  |
| Leuven | 9.25 | 39.71 | 14.72 | 50°52'26.17"N  4°41'48.69"E |  |  |
| Mechelen | 34.43 | 13.45 | 32.90 | 26.71 | 51°3'15.43"N  4°26'40.65"E |  |
| Oudenaarde | 76.64 | 55.18 | 89.19 | 75.94 | 62.51 | 50°50'45.78"N  3°36'57"E |

**S2 – TPCs for egg development time and size at hatching**

We determined egg size (prior to the temperature treatments) by measuring the length of five random eggs per clutch using a digital camera attached to a binocular microscope. To determine size at hatching, we measured the head width of each larva on day 0 (i.e. directly after hatching) using a digital camera attached to a binocular microscope. We defined egg development time as the duration between oviposition and egg hatching.

To assess the effects of urbanization level and rearing temperature on egg size, hatchling size, and egg development rate, we used separate (generalized) linear mixed-effects models. We tested for the effects of urbanization level (urban and rural) and temperature (both linear and quadratic term) on various response variables by including these terms, and their interactions, as fixed effects. As an exception, temperature was not included in the egg size model (egg sizes are fixed and do not change with temperature). In addition, models for egg development time and hatchling body size included egg size as a covariate. Finally, the following random effects were added where appropriate: population, nested within urbanization level (accounting that animals from the same pond are not independent replicates, thereby avoiding pseudoreplication), and female identity of the offspring (accounting for among-brood variation). We provide a detailed summary of the model structures in Appendix S4. We used the R package ‘lme4’ (Bates et al. 2015) for mixed-effects models, and the package ‘car’ to compute Wald χ^2^ statistic and *p*-values for fixed effects (Fox and Weisberg 2011).

**Results**

Egg size did not differ between urbanization levels (χ^2^ = 0.42, d.f. = 1, *P* = 0.52). Egg development time decreased with increasing egg size (χ^2^ = 4.04, d.f. = 1, *P* = 0.04). Increasing temperatures resulted in a four times faster egg development, going from ca. 29 days at 16 °C to ca. 7 days at 30 ° C (linear effect temperature: χ^2^ = 28.72, d.f. = 1, *P* < 0.001) (Fig. S1A). The quadratic effect of temperature was significant as well (χ^2^ = 7.65, d.f. = 1, *P* = 0.01), indicating the temperature effect was strongest at the lower temperatures (Fig. S1A). The quadratic TPC for egg development time did not differ between urban and rural eggs (Temperature × Urbanization level: χ^2^ = 0.001, d.f. = 1, *P* = 0.98; Temperature² × Urbanization level: χ^2^ = 0.003, d.f. = 1, *P* = 0.95), neither was there a fixed difference in development time between urban and rural eggs (χ2 = 1.83, d.f. = 1, *P* = 0.18).

Hatchling body size (measured as larval head width at hatching) was affected by temperature, with intermediate temperatures resulting in a greater body size (linear effect of temperature: χ^2^ = 61.11, d.f. = 1, *P* < 0.001, quadratic effect of temperature: χ^2^ = 71.53, d.f. = 1, *P* < 0.001, Fig. S1B). The quadratic TPC for hatchling size did not differ between urban and rural larvae (Temperature × Urbanization level: χ^2^ = 0.18, d.f. = 1, *P* = 0.67; Temperature² × Urbanization level: χ^2^ = 0.18, d.f. = 1, *P* = 0.67), neither was there a fixed difference in initial size between urban and rural larvae (χ^2^ = 2.60, d.f. = 1, *P* = 0.11). Egg size did not have a significant effect on hatchling body size (χ^2^ = 1.82, d.f. = 1, *P* = 0.18).

**Discussion**

While egg development time and hatchling size showed clear thermal responses, this was not associated with thermal adaptation. As observed in many other taxa (Gillooly et al. 2002; including aquatic insects: Pritchard et al. 1996; Gillooly and Dodson 2000) increasing temperatures shortened the egg development time. This has been explained by the metabolic theory of ecology: due to the thermodynamics of enzyme-catalysed reactions, biological rates are predicted to increase with temperature (Gillooly et al. 2001; Brown et al. 2004). This may also have contributed to the decline in hatchling size at higher temperatures, which was previously observed in the study species by Van Doorslaer and Stoks (2005). This apparent trade-off between egg development times and hatchling size has been described before in various taxa, including insect species (e.g., hawkmoth: Potter et al. 2011; a tropical butterfly: Geister et al. 2009). These studies have argued that the mechanisms underlying the so-called temperature-size rule, where high developmental temperatures result in small adult size (Atkinson 1994), apply to embryonic development as well. The biophysical model by van der Have and De Jong (1996) suggests that high temperatures accelerate cell division, while the increase in cellular growth does not catch up with the cell division rate. This may be a possible explanation for larger hatchlings at lower temperatures.

**Figure S1**. Thermal performance curves for (A) egg development time and (B) size at hatching of urban and rural populations of the damselfly *Coenagrion puella*. Egg development time was calculated as the time between oviposition and hatching. Size at hatching was measured as head width of newly emerged larvae. Least-square means ± 1 SE are shown.

**References**

Atkinson, D. 1994. Temperature and organism size – a biological law for ecototherms? Advances in Ecological Research 25:1–58.

Bates, D., M. Mächler, B. Bolker, and S. Walker. 2015. Fitting linear mixed-effects models using lme4. Journal of Statistical Software 67:1–48.

Brown, J. H., J. F. Gillooly, A. P. Allen, V. M. Savage, and G. B. West. 2004. Toward a metabolic theory of ecology. Ecology 85:1771–1789.

Fox, J., and S. Weisberg. 2011. An {R} companion to applied regression, 2nd ed. Sage, Thousand Oaks, California.

Geister, T. L., M. W. Lorenz, K. H. Hoffmann, and K. Fischer. 2009. Energetics of embryonic development: effects of temperature on egg and hatchling composition in a butterfly. Journal of Comparative Physiology B 179:87–98.

Gillooly, J. F., J. H. Brown, G. B. West, V. M. Savage, and L. Eric. 2001. Effects of size and temperature on metabolic rate. Science 293:2248–2251.

Gillooly, J.F., E. L. Charnov, G. B. West, V. M. Savage, and J. H. Brown. 2002. Effects of size and temperature on developmental time. Nature 417:70–73.

Gillooly, J. F., and S. I. Dodson. 2000. The relationship of egg size and incubation temperature to embryonic development time in univoltine and multivoltine aquatic insects. Freshwater Biology 44:595–604.

Potter, K. A., G. Davidowitz, and H. A. Woods. 2011. Cross-stage consequences of egg temperature in the insect *Manduca sexta*. Functional Ecology 25:548–556.

Pritchard, G., L. D. Harder, R. A. Mutch, and A. Robert. 1996. Development of aquatic insect eggs in relation to temperature and strategies for dealing with different thermal environments. Biological Journal of the Linnean Society 58:221–244.

van der Have, T.M., and G. de Jong. 1996. Adult size in ectotherms: temperature effects on growth and differentiation. Journal of Theoretical Biology 183:329–340.

Van Doorslaer, W., and R. Stoks. 2005. Thermal reaction norms in two *Coenagrion* damselfly species: contrasting embryonic and larval life-history traits. Freshwater Biology 50:1982–1990.

**S3 – Complementary growth rate analyses**

We could not directly determine increases in body mass as larvae are too fragile to handle during early stages. To assess whether the head width measurements also reflected patterns in body mass, we quantified the correlation of head width and wet body mass at the end of the experiment (day 50). At that moment, all larvae were weighed to the nearest 0.01 mg after gently blotting them dry with tissue paper before weighing to ensure that no water remained on the larva. Head width and wet mass measured at day 50 were highly correlated (*r* = 0.94, *P* < 0.001).

Given that comparisons of growth patterns may be confounded with size differences in case of size dependent growth rates, we took special precautions based on Tammaru and Esperk (2007) and Nicieza and Álvarez (2009). When analysing growth rate, we included larval size at hatching as a covariate to the model (see main text). In addition to this, we also ran a related model with a time-varying size-related covariate: size at hatching for growth during the first period, and the size at the start of the second period for growth during the second period. Moreover, we ran a separate model with a repeated-measures design using the subsequent sizes of a given larva (at days 0, 30 and 50) as repeated response variable (instead of the two growth rates) (Nicieza and Álvarez 2009). Population (nested within urbanization level) and female identity of the offspring was included as random effects to all models. We used the R package ‘lme4’ for constructing (generalized) linear mixed models (Bates et al. 2015) and the ‘car’ package to compute Wald χ^2^ statistic and *p*-values for fixed effects (Fox and Weisberg 2011).

**Results and discussion**

The finding of the period-dependent urbanization effect on growth rate during the second period (see Results in main text) did not change qualitatively when including size as time-varying covariate (Urbanization level × Growth period: χ^2^ = 8.96, d.f. = 1, *P* = 0.003), or when using size as response variable in a repeated-measures model (Urbanization level × Growth period: χ^2^ = 18.8, d.f. = 2, *P* < 0.001) indicating this pattern is no artefact of size differences between periods.

**References**

Bates, D., M. Mächler, B. Bolker, and S. Walker. 2015. Fitting linear mixed-effects models using lme4. Journal of Statistical Software 67:1–48.

Fox, J., and S. Weisberg. 2011. An {R} companion to applied regression, 2nd ed. Sage, Thousand Oaks, California.

Nicieza, A. G., and D. Álvarez. 2009. Statistical analysis of structural compensatory growth: how can we reduce the rate of false detection? Oecologia 159:27–39.

Tammaru, T., and T. Esperk. 2007. Growth allometry of immature insects: larvae do not grow exponentially. Functional Ecology 21:1099–1105.

**S4 – Structure of mixed-effect models**

**Table S2.** Model structures used for testing effects of urbanization level and temperature on various response variables. Error structure, fixed effects, covariates, and random effects used in the models are shown separately for each response variable. Note that in all cases except for egg size, both the linear and quadratic term of temperature was included as fixed effects. Urb = urbanization level, Temp = temperature, Period = growth period.

| **Response variable** | **Error structure**  **(function)** | **Fixed effects** | **Covariate** | **Random effects** |
| --- | --- | --- | --- | --- |
| Growth rate | Normal  (identity link) | Urb, Temp, Period,  Urb × Temp,  Urb × Period, Temp × Period,  Urb × Temp × Period | Hatchling size | Population + Female ID + Individual ID |
| Survival | Binomial  (logit-link) | Urb, Temp,  Urb × Temp |  | Population + Female ID |
| Egg size | Normal  (identity link) | Urb |  | Population + Female ID |
| Hatchling size | Normal  (identity link) | Urb, Temp,  Urb × Temp | Egg size | Population + Female ID |
| Egg development rate | Poisson  (log-link) | Urb, Temp,  Urb × Temp | Egg size | Population + Female ID |

**S5 – TMV parameters**

**Table S3.** Parameters obtained from the TMV method. w = width (dimensionless); m (T_opt_) = optimal temperature; h = height (day^-1^); Z_max_ = maximum performance (day^-1^). We refer to Izem and Kingsolver (2005) for detailed explanations of the parameters.

| **Urbanization level** | **w** | **m (T_opt_ °C)** | **h** | **Z_max_** |
| --- | --- | --- | --- | --- |
| Rural | 0.9876 | 20.138 | 0.0009 | 0.0189 |
| Urban | 1.0113 | 21.636 | -0.0009 | 0.0167 |

**S6 – Testing for the consistency of populations within a given urbanization level**

Aside from including population (nested within urbanization level) as a random effect to each model (see Statistical analyses in main text), we constructed additional models to test whether populations from the same urbanization level were consistent in their response to the temperature gradient. For this, we ran separate models for urban and rural individuals where we included the interaction term of population and temperature (both linear and quadratic term). A lack of significant interactions would indicate the consistency of populations within a given urbanization level. We did this for growth rate during the second period (i.e. day 30-50) and larval survival, as for these response variables a significant urbanization level effect was detected (see Results in main text). These models also included larval size at hatching as a covariate, and female identity of the offspring as a random effect. We used the R package ‘lme4’ for constructing (generalized) linear mixed models (Bates et al. 2015) and the ‘car’ package to compute Wald χ^2^ statistic and *p*-values for fixed effects (Fox and Weisberg 2011).

**Results and discussion**

In terms of growth rate, rural populations did not differ in their response to the temperature gradient (Population × Temperature: χ^2^ = 1.35, d.f. = 2, *P* = 0.51; Population × Temperature²: χ^2^ = 1.64, d.f. = 2, *P* = 0.44), and neither did urban populations (Population × Temperature: χ^2^ = 4.94, d.f. = 2, *P* = 0.08; Population × Temperature²: χ^2^ = 4.47, d.f. = 2, *P* = 0.11).

Analysis of larval survival revealed that urban populations did not differ in their response to the temperature gradient (Population × Temperature: χ^2^ = 5.3, d.f. = 2, *P* = 0.07; Population × Temperature²: χ^2^ = 5.6, d.f. = 2, *P* = 0.06). We found a significant interaction between rural populations and the linear (χ^2^ = 14.2, d.f. = 2, *P* < 0.001) and quadratic term of temperature (χ^2^ = 15.8, d.f. = 2, *P* < 0.001), indicating that populations from rural ponds differed in their response to the temperature gradient. Visual inspection of Figure 3 (main text) suggested this was because of the populations showing considerable variation in survival at 30 °C. At the set of other temperatures, there were no significant interactions between population and the linear or quadratic term of temperature (rural populations: Population × Temperature: χ^2^ = 0.94, d.f. = 2, *P* = 0.62; Population × Temperature²: χ^2^ = 0.92, d.f. = 2, *P* = 0.63; urban populations: Population × Temperature: χ^2^ = 1.99, d.f. = 2, *P* = 0.37; Population × Temperature²: χ^2^ = 1.97, d.f. = 2, *P* = 0.37), indicating that for this temperature range populations from the same urbanization level were consistent in their response to the temperature gradient.

**References**

Bates, D., M. Mächler, B. Bolker, and S. Walker. 2015. Fitting linear mixed-effects models using lme4. Journal of Statistical Software 67:1–48.

Fox, J., and S. Weisberg. 2011. An {R} companion to applied regression, 2nd ed. Sage, Thousand Oaks, California.

**S7 – Assessing the influence of random effects**

To assess the influence of the random effects population and family, we compared the marginal R^2^, the proportion of variance explained by the fixed factors only, and the conditional R^2^, the proportion of variance explained by both the fixed and random factors (see e.g. Peay et al. 2015). Marginal and conditional R^2^ values were calculated based on Nakagawa and Schielzeth (2013) for mixed-effects models. We used the R package ‘piecewiseSEM’ to obtain marginal and conditional R^2^ values (Lefcheck, 2015).

Fixed effects alone explained 88.76 % of the variance in the growth model (marginal R^2^), whereas the variance explained by both the fixed and random effects was 88.82 % (conditional R^2^); indicating that the inclusion of the random effects (i.e. population nested within urbanization level and identity of the larvae nested within the female identity) did not increase the explanatory power.

As in the growth rate model, the inclusion of the random effects (i.e. population nested within urbanization level and identity of the female) resulted in a negligible increase in explanatory power: fixed effects alone explained 21.41 % of the variance in the survival model (marginal R^2^), whereas the variance explained by both the fixed and random effects was 21.69 % (conditional R^2^). This also indicates that any differences in the response of the populations of the same urbanization level were minor.

**References**

Lefcheck, J.S. 2015. piecewiseSEM : Piecewise structural equation modeling in R for ecology, evolution, and systematics. Methods in Ecology and Evolution 7:573–579.

Nakagawa, S., and H. Schielzeth. 2013. A general and simple method for obtaining R^2^ from generalized linear mixed-effects models. Methods in Ecology and Evolution 4:133–142.

Peay, K. G., S. E. Russo, K. L. McGuire, Z. Lim, J. P. Chan, S. Tan, S. J. Davies. 2015. Lack of host specificity leads to independent assortment of dipterocarps and ectomycorrhizal fungi across a soil fertility gradient. Ecology Letters 18:807–816.

**S8– Predator communities**

Given that differences in predator densities may contribute to differences in growth rate (e.g. Laurila et al. 2008), we estimated the densities of predators feeding on damselfly larvae. To this end, we conducted dipnet sweeps (3 m haul distance) at five different locations in each pond using a D-frame dipnet (0.5 mm mesh size) on 7 October 2016. The five locations per pond were selected based on the presence of submerged aquatic vegetation where damselfly larvae and their predators frequently occur. Predator densities were presented as the sum of the five samplings per pond. Differences in predator community composition between urban and rural ponds were visualized using non-metric multidimensional scaling. We compared total predator densities of urban and rural ponds with a t-test.

Although predator density differed between ponds (Table S3), this was not consistent among populations from the same urbanization level (*t* = 0.987, d.f. = 2.044, *P* = 0.426). Similarly, ponds had distinct predator assemblages, yet they did not cluster based on their urbanization level (Fig. S2).

**References**

Corbet, P. S. 1999. Dragonflies: behavior and ecology of Odonata. Cornell University Press. UK.

Laurila, A., B. Lindgren, and A. T. Laugen. 2008. Antipredator defenses along a latitudinal gradient in *Rana temporaria*. Ecology 89:1399–1413.

McPeek, M. A. 1990. Behavioral differences between *Enallagma* species (Odonata) influencing differential vulnerability to predators. Ecology 71:1714–1726.

Siepielski, A. M., K. L. Hung, E. E. B. Bein, and M. A. McPeek. 2010. Experimental evidence for neutral community dynamics governing an insect assemblage. Ecology 91:847–857.

**Table S4**. Community and densities of damselfly predators at each of the six study ponds. Numbers per pond are the sum of the five samples per pond. Taxa were categorized as damselfly predators based on McPeek 1990; Corbet 1999; Siepielski et al. 2010.

|  | Rural populations | | | Urban populations | | |
| --- | --- | --- | --- | --- | --- | --- |
|  | Bierbeek | Bornem | Houwaart | Leuven | Mechelen | Oudenaarde |
| Anisoptera | 8 | 0 | 1 | 0 | 4 | 0 |
| *Ranatra linearis* | 6 | 0 | 0 | 0 | 1 | 0 |
| Notonectidae | 21 | 56 | 20 | 0 | 11 | 0 |
| Nepidae | 2 | 0 | 1 | 0 | 0 | 0 |
| Dytiscidae | 16 | 7 | 8 | 0 | 44 | 0 |
| Sialidae | 0 | 0 | 24 | 0 | 27 | 0 |
| Newts | 0 | 0 | 0 | 5 | 0 | 0 |
| Sticklebacks | 0 | 0 | 6 | 0 | 0 | 0 |
| **Total** | 53 | 63 | 60 | 5 | 87 | 0 |

**Figure S2**. Non-metric multidimensional scaling of damselfly predator communities among the six study ponds. Urban (red) and rural (green) pond codes are given in Table S1.
